# Management of Complex Acetabular Fractures by Using 3D Printed Models

**DOI:** 10.3390/medicina58121854

**Published:** 2022-12-15

**Authors:** Stoyan Ivanov, Petar Valchanov, Stoyan Hristov, Deyan Veselinov, Boyko Gueorguiev

**Affiliations:** 1 Department of Orthopaedics and Traumatology, Medical University of Varna, 9002 Varna, Bulgaria; 2 Department of Anatomy, Histology and Embryology, Medical University of Varna, 9002 Varna, Bulgaria; 3 Department of Orthopedics and Traumatology, University Hospital for Active Treatment, 8018 Burgas, Bulgaria; 4 Bulgarian Academy of Sciences, Institute of Metal Science ‘Acad. A. Balevski’, 1574 Sofia, Bulgaria; 5 AO Research Institute Davos, 7270 Davos, Switzerland

**Keywords:** 3D printed models, complex acetabular fractures, implant selection, surgical treatment

## Abstract

*Background and Objectives*: Using 3D printed models in orthopaedics and traumatology contributes to a better understanding of injury patterns regarding surgical approaches, reduction techniques, and fracture fixation methods. The aim of this study is to evaluate the effectiveness of a novel technique implementing 3D printed models to facilitate the optimal preoperative planning of the surgical treatment of complex acetabular fractures. *Materials and Methods*: Patients with complex acetabular fractures were assigned to two groups: (1) conventional group (n = 12) and (2) 3D printed group (n = 10). Both groups included participants with either a posterior column plus posterior wall fracture, a transverse plus posterior wall fracture, or a both-column acetabular fracture. Datasets from CT scanning were segmented and converted to STL format, with separated bones and fragments for 3D printing in different colors. Comparison between the two groups was performed in terms of quality of fracture reduction (good: equal to, or less than 2 mm displacement, and fair: larger than 2 mm displacement), functional assessment, operative time, blood loss, and number of intraoperative x-rays. *Results*: A significant decrease in operative time, blood loss, and number of intraoperative x-rays was registered in the 3D printed group versus the conventional one (*p* < 0.01), with 80% of the patients in the former having good fracture reduction and 20% having fair reduction. In contrast, 50% of the patients in the conventional group had good reduction and 50% had fair reduction. The functional score at 18-month follow-up was better for patients in the 3D printed group. *Conclusions*: The 3D printing technique can be considered a highly efficient and patient-specific approach for management of complex acetabular fractures, helping to restore patient′s individual anatomy after surgery.

## 1. Introduction

Acetabular fractures are disabling injuries with vast psychological and functional burdens to the patients. Their rate has been increasing over the years, affecting both patients in active age and elderly patients [1,2]. Frequently, associated injuries affecting the head, chest, or abdominal region accompany these fractures [3]. All acetabular fractures require surgical treatment to ensure congruence of the hip joint and stability; therefore, anatomical reduction and stable fixation are prerequisites for an optimal and functional outcome [4,5].

The complex three-dimensional (3D) osseous anatomy of the pelvic ring—accompanied by a lot of nerves and major blood vessels—and the necessity for application of both indirect reduction techniques and surgically demanding exposures make acetabular fractures challenging, even for experienced orthopaedic trauma surgeons. These complex injuries are characterized by presence of various fracture lines and using only conventional radiography and axial computed tomography (CT) scanning makes their classification, intraoperative spatial orientation, and surgical treatment demanding [6,7]. Even with the use of 3D multiplanar CT reconstructions, acetabular fractures are difficult to characterize and analyze preoperatively because the volume-rendered models are visualized on a conventional two-dimensional (2D) screen [8,9]. Recently, with the development of digital technologies, new methods including the use of 3D printed models are increasingly applied for better outcomes and enhanced safety of the procedures [10]. 

Associated acetabular fractures are characterized by two or more fracture lines with complex geometries passing through the acetabulum [11]. The creation of preoperative 3D models allows to (1) get a real impression about the injury severity, (2) avoid intraoperative complications, (3) utilize an optimal surgical approach, (4) use limited exposures, (5) select an appropriate implant, and (6) decide on its optimal positioning. The exploration of pelvic soft tissues can be trained through dissections during cadaveric courses or studied in the literature; however, the occasions for analysis of acetabular fracture types are limited. Their characteristic features make them appropriate to evaluate the efficiency of using 3D printing techniques. Therefore, the aim of the present study is to evaluate the effectiveness of a novel technique implementing 3D printed models to facilitate the optimal preoperative planning of the surgical treatment of complex acetabular fractures.

## 2. Materials and Methods

This level 1 retrospective cohort study was conducted from September 2018 to December 2021, in line with the principles of the Declaration of Helsinki. Approval was granted by the local Ethics Committee (NTTMV-16087/28 September 2018). Informed consent was obtained from all participants. Patients with complex acetabular fractures were assigned to two groups: (1) conventional group (10 males and 2 females) and (2) 3D printed group (9 males and 1 female). Fracture patterns were classified according to the Judet–Letournel classification based on standard radiographs and CT scans [12]. Both groups included participants with either a posterior column plus posterior wall fracture, a transverse plus posterior wall fracture, or a both-column acetabular fracture. Patients with simple fractures and a time from injury to surgery longer than 2 weeks were excluded from the study. The 3D printed technique implemented in the procedure of acetabular fracture treatment has been routinely applied in our practice since the beginning of 2020.

### Technical Specifications

**Figure 1:**
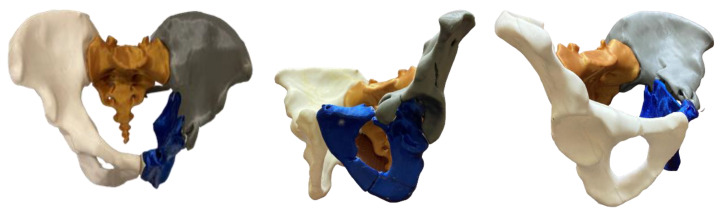
A 3D printed pelvis model of a clinical case with a complex acetabular fracture is illustrated in inlet (**left**), obturator oblique (**middle**), and iliac oblique (**right**) views.

In this study, 3D models were generated from CT scans with slice thicknesses of 1.0 to 1.5 mm. The datasets were presented in a Digital Imaging and Communications in Medicine (DICOM) format, including bone and soft tissue window series. All voxels with Hounsfield units of 150 or higher were segmented using Slicer3D software package [13] and exported as a Standard Triangle Language (STL) file featuring surface rendering. The STL model was edited with Autodesk Meshmixer software [14] and all artifacts were processed via surface modeling. The final model was reimported into Slicer 3D [13], reconverted into a segmentation, and compared with the original datasets visually as a quality assurance technique. When the fidelity of the final model was confirmed, the 3D model was retopologized and prepared for 3D printing by subtracting both femoral heads. The 3D printed objects were fabricated with an Original Prusa MK3S 3D printer [15] with a nozzle size of 0.4 mm, using different colors of natural and smooth polylactide (PLA) filament for each separate bone and fractured fragment for better differentiation and representation (Figure 1). The layer height was 0.15 mm, with a 15% infill, 4 external shells, and some support material for the overhanging parts of the structures. Depending on the size of the model, the 3D printing itself took 48–54 h.

**Figure 2:**
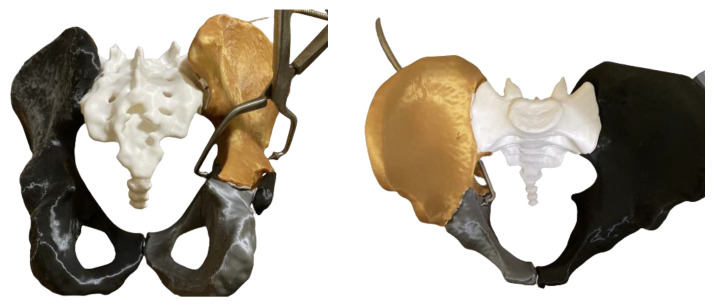
Provisional reduction and placement of the asymmetric clamp through the greater sciatic notch are illustrated in posterior (**left**) and top (**right**) views.

The preoperative 3D printed models provided 360° visualization of the actual size of the patient’s pelvis, thus determining the fracture pattern and ensuring the tactile perception of the fragments’ size and displacement. Optimal provisional reduction was performed and the necessity of using special clamps during surgery was evaluated (Figure 2).

**Figure 3:**
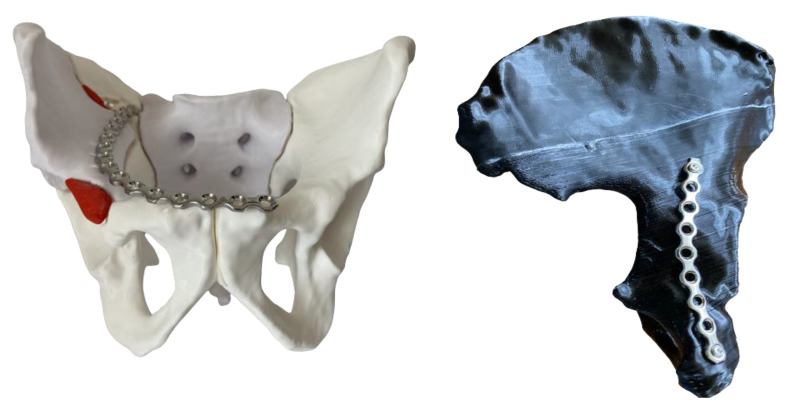
A 3D printed pelvis model of a patient with a preoperatively contoured anterior column plate, placed after fracture reduction, with no fixation of the iliac ring (**left**); a 3D printed model of a hemipelvis from another clinical case, generated via mirroring of the uninjured contralateral side, and visualized with a posterior column plate with temporary fixation (**right**).

Appropriate plate configuration was selected and preoperative contouring of the implants was performed. All implants were then sterilized and prepared for use. The length of the screws and their insertion directions were determined. Intraoperatively, these implants needed a small adjustment to fit on the bone surface (Figure 3).

**Figure 4:**
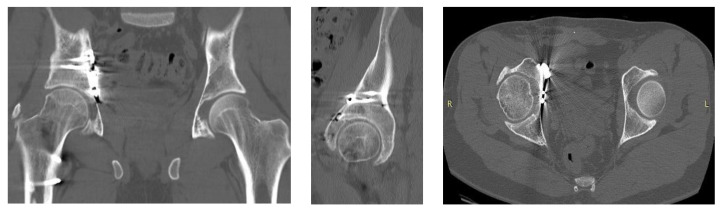
Postoperative CT scan of a clinical case exhibited in coronal (**left**), sagittal (**middle**), and axial (**right**) views and demonstrating excellent anatomical reduction.

Depending on the fracture pattern, the surgical approaches utilized in both groups were the anterior intrapelvic modified Stoppa approach—in selected cases with the addition of the lateral window of the ilioinguinal approach—and the posterior Kocher–Langenbeck approach. Intraoperatively, fracture reduction was assessed in anteroposterior (AP) and Judet oblique views. Postoperative CT scanning was performed to evaluate hip joint congruency and to verify the positions of the plates and screws (Figure 4).

**Figure 5:**
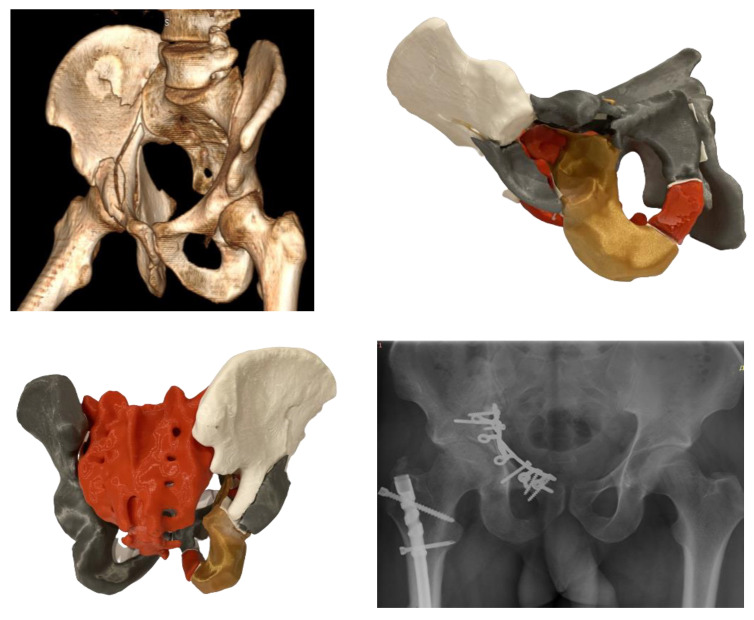
A reconstructed CT scan from a clinical case with substantial comminution of the quadrilateral surface (**top left**). A corresponding 3D printed pelvis model in (1) inside view, with extracted femoral head for better visualization of the intra-articular acetabular surface (**top right**), and (2) posterior view (**bottom left**), demonstrating comminution of both the posterior column and posterior wall. A postoperative radiographic image of the fixation with an omega plate (**bottom right**).

The anatomical reduction was considered as achieved when the articular surface was restored within a 2 mm step. Mostly, 3.5 mm reconstruction titanium pelvic plates were used for fracture fixation in combination with a 1/3 tubular titanium plate (DePuy Synthes, Zuchwil, Switzerland). However, in selected cases with substantial comminution of the quadrilateral surface, a specially designed omega plate (Medin, Nové Město, Czech Republic) was used for fixation through the intrapelvic anterior approach (Figure 5): first, a regular CT scan of the patient was obtained with 3D reconstruction; second, based on this, a 3D printed model was created for a detailed presentation of the multiple fracture lines on the intra-articular acetabular surface, with a detachment of the anterior and posterior columns; third, the 3D printed model was used for analysis of the fracture morphology, provisional reduction of the printed fragments, as well as for selection of the plate size and its precontouring.

Routine antibiotic and anticoagulation prophylactics were obtained and early continuous passive motion exercises for ankle, knee, and hip joints were conducted. A non-weight bearing period of at least 8 weeks was recommended, followed by 30% to 50% partial weight bearing, depending on the fracture pattern, patient’s weight, and compliance.

The comparative characteristics between the two groups included: (1) quality of reduction (good: equal to, or less than 2 mm displacement, and fair: larger than 2 mm displacement) according to previously published work [16,17,18]; (2) functional assessment (modified Merle d’Aubigne–Postel score); (3) operative time; (4) blood loss; (5) number of intraoperative x-rays; (6) complications.

Statistical analysis was performed using the SPSS software package (V.27, IBM SPSS, Armounk, NY, USA). A variational descriptive analysis of all quantitative variables was performed to calculate the corresponding mean values, standard deviations (SDs), medians, and ranges. Mann–Whitney, Wilcoxon Signed-Rank, and Chi Square tests were performed to screen and detect significant differences between the groups. Level of significance was set to 0.05 for all statistical tests.

## 3. Results

Gender and fracture type distributions in the study groups are presented in Table 1.

The most common fracture pattern was represented by a both-column fracture. Fractures in other anatomical regions (femur and distal radius) were present in 22.7% of the cases. 

Further parameters of interest in the study groups are presented in Table 2. Patients’ age in the conventional and 3D printed groups was 50.9 and 49.1 years on average, respectively, with no significant difference between the groups (*p* = 0.74). The time from injury to operation in the conventional and 3D printed groups was 7.5 and 7.8 days on average, respectively, with no significant difference between the groups, and no significant influence on the outcome (*p* ≥ 0.56). The mean follow-up periods in the conventional and 3D printed groups were 17.6 and 17.1 months, respectively, with no significant difference between them (*p* = 0.78). There was a significant difference between the groups in terms of operative time, blood loss, and number of intraoperative x-rays, in favor of the 3D printed group (*p* < 0.01). The modified Merle d’Aubigne–Postel score at the follow-up was significantly better for the patients in the 3D printed group (*p* = 0.03). 

A second functional assessment, performed in the conventional study group after 35.5 months on average (range 34–36 months), revealed a mean modified Merle d’Aubigne–Postel score of 12.6 (SD 2.0; median 13; range 9–15), which was significantly worse compared to the functional score for the first follow-up period (*p* = 0.03).

Regarding the quality of the reduction, 50% of the patients in the conventional group had a good reduction and 50% had a fair reduction, whereas in the 3D printed group 80% of the patients demonstrated a good reduction and 20% a fair one. However, this difference between the groups was not significant (*p* = 0.14).

Superficial surgical infection was observed in one patient from the 3D printed group and two patients from the conventional group. Secondary arthritis due to avascular necrosis was observed in one patient from the conventional group. Other complications such as nerve palsy, heterotopic ossification, or loss of reduction did not occur in any group.

## 4. Discussion

The surgical treatment of acetabular fractures is a demanding hazardous procedure requiring an experienced surgical team [19,20]. Meticulous soft tissue dissection and detailed knowledge of anatomy are crucially important. An optimal surgical approach, reduction techniques, implant selection and placement should be determined prior to surgery to achieve good outcomes [21]. The use of physical 3D printed models improves the intraoperative spatial orientation and understanding of the complex anatomy compared to 3D CT scan reconstructions [22]. This novel technical trend requires comprehensive cooperation between medical engineers and medical staff [23,24].

The 3D printing technique helps understand and reliably classify complex acetabular fractures, thereby ensuring more accurate reduction and fixation [25]. The 3D models offer full visualization and, at the same time, palpation of the fragments’ size and physical assessment of their displacement direction. These models can be used for precontouring of implants, thereby increasing the precision and safety of the procedure, and are extremely beneficial for inexperienced surgeons [26,27]. Several studies focus on the application of 3D printing for management of complex acetabular fractures and emphasize the benefits of better fracture pattern understanding and reduced operative time [16,28,29].

In the present study, an average decrease of 59 min in the surgical duration was indicated in favor of the 3D printed group. Similar results were demonstrated when comparing the 3D printed and control groups in a meta-analysis based on four randomized control trials and nine retrospective studies with a mean of 38.8 min of less operational time in the 3D printed group [30,31]. Some authors even reported a difference in surgical time of 70 min to the detriment of the conventional group [17]. The shorter operation time is associated with improved outcomes, reduced blood loss, and a lower rate of infectious complications. Information on blood loss, extracted from the anesthesiologists’ papers, indicates a mean decrease of 172.5 mL in the 3D printed group. Our results are consistent with the data from nine retrospective studies, where 225.1 mL less blood loss was registered in the 3D printed group versus the conventional one [31].

In acetabular fracture treatment, three disease-specific outcome scores are mainly used: the Merle d’Aubigné and Postel score, the modified Merle d’Aubigné–Postel score (Matta et al.), and the Harris hip score [32,33]. The postoperative functional recovery in our study was significantly better in the 3D printed group than in the conventional group at the 18-month follow-up. Five studies reported similar results, where the control group was likely to achieve fewer good/excellent results than the 3D printed group [31]. This may be due to the less invasive dissection performed in the latter. The functional evaluation at 36 months after surgery revealed a decline in the results of the conventional group due to osteoarthritis.

The quality of the reduction was assessed intraoperatively via AP x-rays and oblique (Judet) projections, and postoperatively by CT scanning. Patients in the conventional group were less likely to achieve good and excellent reduction than in the 3D printed group. Many retrospective studies and several randomized control trials reported similar results [31,34]. However, acetabular surgery is still more experience-based surgery. Precisely, the digital development of 3D printed models gives an opportunity to shorten the so-called “learning curve”.

The use of modern 3D printed acetabular models in fracture management allows for reduction of intraoperative x-ray radiation thus benefitting both patients and intraoperative staff [27,35,36,37]. Our study also demonstrated a significant decrease in the number of intraoperative x-rays. Currently, only one study has reported no significant difference in radiation exposure during surgery [38].

Treatment of complex acetabular fractures often requires extensive approaches or dual incisions. Anticipated complications associated with extensive acetabular approaches are infection, muscle weakness, heterotopic ossifications, nerve injuries, and increased blood loss. An option to minimize these risks is the use of 3D printed models pre- and intraoperatively, as this method allows for detailed inspection of the fracture pattern and selection of single non-extensive or limited invasive surgical exposure, which utilizes indirect reduction techniques. Several studies demonstrated that 3D printing techniques reduce postoperative complications [16,18,39].

The need for intraoperative 3D implant contouring to the sophisticated osseous anatomy and the limited field of view following the surgical approaches make acetabular fractures challenging, even for skilled surgeons [40,41]. The valuable option of using 3D printed models offers the opportunity to shape plates preoperatively in order to reduce surgical time and ensure a better fit of the implant to the bone [42]. This is extremely helpful and makes the procedure safer in patients with obesity and complex fractures treated only via an anterior approach where the implants are located beneath the external iliac vessels. The 3D models are extremely useful to ensure safer screw placement in cases where percutaneous or column screws are used for indirect fixation. Moreover, these precise 3D physical models facilitate the decision for optimal plate configuration around the pelvic brim—a suprapectineal plate in combination with a horizontal infrapectineal plate or a 1/3 tubular plate as a vertical infrapectineal plate. Last but not least, acetabular surgery is time-consuming, may take hours, and plate precontouring reduces the surgeon’s fatigue and preserves concentration during the procedure. De Franco et al. used a 3D printed technique in a case of fracture-related infection after a bicolumnar acetabular fracture with femoral head necrosis [43]. The authors performed a dry surgery on the 3D printed model in order to decide how to reduce the fracture components, obtain a new functional acetabulum, select the appropriate type and size of implants, and the direction of the screws.

Nevertheless, there are several disadvantages of using 3D printed models. First, this technique is relatively expensive and requires a time-consuming procedure, which excludes its use in emergency cases. Second, the accuracy of the printed models is mainly influenced by the scan parameters, and high-quality CT images with a slice resolution of less than 0.7 mm are needed. Third, no information on soft tissue injuries is available (e.g., Morel-Lavallee lesion). Last, 3D printed models should be validated in order to test the accuracy of the 3D printing process. Currently, according to the most popular guideline for medical 3D printing, a visual assessment is considered the gold standard as a quality assurance technique [44]. There are also other techniques for quality assurance, such as measurements of defined distances on the imaging dataset and on the 3D printed object [45].

The present study has several drawbacks, including its retrospective nature and the comparatively small cohort of patients. A strong limitation is the quite short follow-up period of insufficient length for evaluation of the functional results after severe acetabular fractures; moreover, relatively low-quality datasets were used for modeling. The difference between the bone structure of the patients and the 3D printed objects depends mainly on two parameters of the CT scan—slice thickness and kilovoltage peak (kVp). A dataset with a slice thickness of 0.4 mm and kVp of 120 can result in a model with an average deviation of 0.4 mm compared with the source structure; however, the quality drops significantly if a larger slide thickness is used. The datasets used in this study were with a slice thickness of 1 mm and kVp of 120, resulting in a model with an average deviation of ± 1.65 mm [46]. The accuracy of the segmentation can be improved via resampling by decreasing the voxel size, which improves the dimensional accuracy down to ± 0.95 mm. Most of the errors in the conversion of the dataset into a 3D printed model are segmentation-related and can be improved significantly using higher-resolution datasets. The lowest quality of a dataset for modeling of a human bone model is 1 mm slide thickness and a voxel size of 0.5 × 0.5 × 0.5 mm. As a quality control technique, a cadaveric study is performed to validate the workflow used for generation of the models. A cadaver is CT-scanned, converted to a 3D file and 3D printed. After finishing of the model, both the source object from the cadaver and the 3D printed one are measured, and the average difference is calculated [47]. The validation of the workflow is performed once per one or two years; however, since the whole procedure is time-consuming, another technique—visual validation comparison between the CT dataset and the segmentation obtained from it—is used for the individual clinical cases.

Special consideration requires the management of acetabular fractures in elderly patients due to their increased occurrence, severe osteoporosis with lower bone quality, many comorbidities, and significant mortality. With an average increase of acetabular fractures by 23% per year, the surgical treatment will benefit from existing prerequisites for limited invasive surgery assisted by 3D printing technology [48]. The use of 3D printed pelvic models for training purposes demonstrates a consensus among young and experienced surgeons regarding such parameters as fracture type, surgical approach selection and sequence, and patient positioning [25].

Future prospective studies should focus on the 3D printing of implants from titanium or polyether-ether-ketone as a patient-specific approach and development of 3D virtual reality in operating theatres [36,49].

## 5. Conclusions

The 3D printing technique can be considered a highly efficient and patient-specific approach for management of complex acetabular fractures, helping to restore the patient′s individual anatomy after surgery. It offers a better opportunity for selection of the most appropriate surgical approach and implants, and is associated with decreased operative time, less blood loss and lower x-ray radiation.

## Figures and Tables

**Table 1:** Gender and fracture type distributions in the study groups: fracture I—both-column fracture; fracture II—transverse plus posterior wall fracture; fracture III—posterior column plus posterior wall fracture.

Parameter	Group		*p*-Value
	Conventional	3D Printed	
Gender	10 m/2 f	9 m/2 f	0.65
Fracture I	6	5	0.93
Fracture II	2	2	0.94
Fracture III	4	3	0.93

**Table 2:** Parameters of interest between groups 1 (conventional) and 2 (3D printed) in terms of mean value, standard deviation (SD), median, and range, together with the corresponding *p*-values.

Parameter	Group	Mean	SD	Median	Range	*p*-Value
Patents age (years)	1	50.9	11.6	54	27–65	0.74
	2	49.1	12.2	54	26–64	
Time from injury to operation (days)	1	7.5	2.4	7	5–8	0.89
	2	7.8	2.8	7	5–9	
Operative time (min)	1	252.5	30.2	240	220–300	<0.01
	2	193.5	26.0	195	160–240	
Blood loss (mL)	1	837.5	68.0	835	780–980	<0.01
	2	665.0	52.7	690	580–720	
Intraoperative x-rays	1	66.5	6.7	65	58–80	<0.01
	2	25.9	5.6	24.5	20–36	
Follow-up (months)	1	17.6	0.7	18	17–19	0.78
	2	17.1	5.2	17	8–24	
Modified Merle d’Aubigné–Postel score at follow-up	1	13.1	1.7	14	10–15	0.03
	2	14.7	1.6	15	12–17	

## Data Availability

The datasets analyzed during the current study are available from the corresponding author upon reasonable request. In order to comply with the requirements of the Ethics Committee, the image set is not available for request due to data privacy policies.
